# A comprehensive investigation of the Galilean moon, Io, by tracing mass and energy flows

**DOI:** 10.1007/s10686-021-09768-y

**Published:** 2021-07-05

**Authors:** N. Thomas

**Affiliations:** Physikalisches Institut, University of Bern, Sidlerstrasse 5, CH-3012 Bern, Switzerland

**Keywords:** lo, Mission, Geophysics, Jupiter system, Magnetosphere, Interactions

## Abstract

Io is the most volcanically-active object in the solar system. The moon ejects a tonne per second of sulphur-rich gases that fill the vast magnetosphere of Jupiter and drives million-amp electrical currents that excite strong auroral emissions. We present the case for including a detailed study of Io within Voyage 2050 either as a standalone mission or as a contribution to a NASA New Frontiers mission, possibly within a Solar System theme centred around current evolutionary or dynamical processes. A comprehensive investigation will provide answers to many outstanding questions and will simultaneously provide information on processes that have formed the landscapes of several other objects in the past. A mission investigating Io will also study processes that have shaped the Earth, Moon, terrestrial planets, outer planet moons, and potentially extrasolar planets. The aim would be simple – tracing the mass and energy flows in the Io-Jupiter system.

## Introduction

Based on the last 10 years, ESA’s science directorate can expect to launch 4–5 missions to Solar System targets within the 2033–2050 timeframe (not including Mission of Opportunity contributions to missions of other agencies and other directorates such as the Human and Robotic Exploration (HRE) directorate). Several mission concepts are regularly discussed as possible future missions including an Ice Giant orbiter, sample return missions to cometary nuclei, the Moon or Mars, a Mars polar rover or orbiter, a Venus atmospheric probe, contributions to NASA missions to Titan and Enceladus, and asteroid/main-belt comet landers. All these ideas have merit. Other missions related to solar-terrestrial physics will also be competitive for the open slots.

Although life detection and potentially habitable objects in our Solar System and other solar systems will remain focal points for ground-based observatories and space-borne observatories and missions throughout the next 30 years, there are still objects with definitively no potential for habitability in our Solar System that can reveal much about physical processes that have influenced the evolution of many Solar System bodies. Furthermore, these objects play to the strengths of the European community because of the community’s efforts in combining magnetospheric physics (based around missions such as Cluster) with planetary physics (based around missions such as Mars Express) in “joint” missions such as Rosetta [19] and BepiColombo [4].

In this document we wish to emphasize that there is one object in our Solar System that would produce enormous excitement for both the magnetospheric and the planetary physics communities and continue the inter-disciplinary approach. That object is Io.

The prediction and identification of volcanic activity from the innermost Galilean satellite, Io, was one of the defining moments of the Voyager exploration of the outer Solar System [45, 47]. Subsequent investigations, using data from the Voyager, Galileo, New Horizons, and Juno missions as well as ground-based and Earth-orbiting observations, have shown that Io and its interactions with the other Galilean satellites and Jupiter’s magnetosphere, are not merely remarkable but the processes acting in this complex system pose an intellectual challenge. Furthermore, while Io itself is totally devoid of water and is one of the most inhospitable places in our Solar System (from thermal, chemical, and radiation perspectives), its influence on objects that may have harboured life (i.e. Europa and Ganymede) are of great significance. In addition, its interactions with Jupiter may provide a model for processes occurring in exoplanet systems. We address here the major scientific goals that a potential mission to Io can resolve. We then look at the principal challenges in implementation.

## Scientific justification

The most recent comprehensive reviews of the processes connected with Io can be found in the relevant chapters of “Jupiter: The Planets, The Satellites and the Magnetosphere” edited by Bagenal et al. from 2004. Each chapter concludes with a series of open questions. Advances since the writing of these reviews have occurred and will be referred to below. However, it should be noted that we have now reached a point where detailed high resolution measurements, through in situ experiment and nearby remote-sensing at high temporal cadence, are needed to make a significant step forward in our understanding of the system. We look at each of the major elements in the system in turn.

### Volcanic activity and internal geophysics

Io is unique in the Solar System in that its main internal heat source is not radioactive decay but tidal dissipation [44]. Io, Europa, and Ganymede form the Laplace resonance in which the mean longitudes of the satellites, λ_I,E,G_, are related via the close approximation

1 $$ {\lambda}_I-3{\lambda}_E+2{\lambda}_G\approx \pi $$

Io is in synchronous rotation in a circular orbit about Jupiter but the tidal interaction with Europa leads to a forced eccentricity that results in a forced motion of the tidal bulge raised on Io by Jupiter. The relative motion of the bulge in Io’s frame results in an energy dissipation of 0.6–1.6 10^14^ W through friction [44]. This heat generation drives the observed volcanic activity.

Lainey et al. [36] have claimed that the system is evolving OUT of the Laplace resonance (at the 3 sigma level). Effectively, Io moves inwards, towards Jupiter, and loses more orbital energy by dissipation of solid-body tides raised by Jupiter and by the Laplace resonance interaction than it gains from the exchange of angular momentum with Jupiter’s rotational energy through tidal dissipation in Jupiter. This would be surprising since it would imply we are at a preferred epoch in the system’s history and hence a test of this paper through high precision tracking is of interest.

The dissipation of heat within the body produced by the friction was modelled by, for example, Segatz et al. [55] and subsequently, Hussmann and Spohn [24] looked at coupled thermal-orbital evolution models of Europa and Io as a system. Recent work has shown that in a body periodically strained by tides, heating is far from homogeneous. The spatial distribution of tidal heating depends in a complicated way on the tidal potential and on the internal structure of the body [5]. This has been taken further [60] by looking at lateral dependencies incorporating mantle convection and dependencies on the internal structure. The basic pattern of Io’s surface activity (with strong equatorial volcanic activity) appears to be broadly reproduced by these models. Constraints can also be determined by studying the gravity field and it should be noted that Io’s C_22_ is >50 times larger than that of Callisto. The effects are very large and easily measureable by spacecraft tracking.

The composition of the core is not known but it is assumed to be completely liquid and that the abundant sulphur on the surface is indicative of large amounts of FeS in a core that is between 10 and 20% of the satellite’s radius. As the temperature of the mantle is not known, its composition cannot be constrained but again there are reasons to suppose that silicates are present with Fe/Si ratios in the range 1.3 to 1.5 [57]. Whether or not Io has a permanent, internal magnetic field is unclear [29]. Most planetary dynamos arise when core convection is driven by cooling from the mantle above. Instead, any Io dynamo could be driven by tides. Establishing constraints on the internal structure through sensing any internal field will, in turn, constrain tidal dissipation models.

### Volcanism and surface geomorphology

It is now well known that volcanism modifies the appearance of the surface of Io such that there is no evidence of ANY impact crater on its surface. Galileo observations of the Pele region indicated the rapid re-surfacing that occurs at rates of the order of cm/year confirming estimates from the Voyager era [27]. The re-surfacing is from sedimentation of the material in the volcanic plumes but also from lava. Flood lavas have been an important part of the geologic history of all terrestrial planets (including the Moon and BepiColombo’s target, Mercury) but are active today only on Io [28] making this a key area of interest. The lava flows can be immense. There are many unanswered questions about the volcanism. The effects of variables such as size, erupted volume, peak effusion rate, episode duration, cooling rate, and atmospheric pressure are largely unknown.

The surface colours are indicative of high sulphur content with the colour changing with temperature [53]. However, there are structures on the surface that are 6–8 km high. This requires a structural strength that pure sulphur cannot support and hence this is indirect evidence of silicates. There remains significant uncertainty about the exact nature of the volcanic material and indeed whether there are different types of volcanism (silicate v. sulphur) present on Io. Categorization of short-lived, but high eruption plumes and longer-lived but small eruption plumes has been made since the Voyager fly-bys. It is necessary to determine the temperature of the erupting lavas as this constrains lava composition [11]. Ground-based observations [13] indicate the complexity of some of the lava-related phenomena.

### The atmosphere and volcanic interactions

The nature of Io’s atmosphere has been the subject of debate for over 40 years. The atmosphere is mostly composed of SO_2_ [43] but this is also one of the gases which drives the volcanic activity. While the volcanoes are the ultimate source of atmospheric gas, it is not clear whether the volcanoes themselves are the main source or whether species on the surface are the main source through a condensation/sublimation cycle. The detection of gaseous SO_2_ by the Voyager IRIS experiment [48] suggested surface densities at the 10^−7^ bar level and therefore collisionally thick. The possibility of having an atmosphere in equilibrium with surface SO_2_ was suggested. However, the SO_2_ detection was close to the volcanic vent, Loki, and subsequent attempts to demonstrate consistency with other observations from Earth have not produced an unambiguous result. Doppler measurement of SO emission at microwave wavelengths [37] and HST measurements of gases above the volcanic vents [42] suggested that much of the “atmosphere’” is of direct volcanic origin. Feaga et al. [17] suggested that the sunlit SO_2_ atmosphere is temporally stable on a global scale while Jessup and Spencer [25] made observations consistent with longitudinal asymmetries and concluded that 20–30% of the gas density is of volcanic origin (cf. [51]).

Because of the strong dependence of SO_2_ equilibrium vapour pressure on temperature, a global atmosphere dominated by an equilibrium between surface SO_2_ ice and the gas phase should collapse when Io enters eclipse behind Jupiter. Tsang et al. [65] observed a factor of 5 decrease in SO_2_ column density during eclipse at 19 μm. However, this contradicted a previous paper by Tsang et al. [64] using HST in the UV where they saw little evidence of atmospheric collapse. In other words, we have very little understanding of the spatial and temporal variation of the gas distribution above Io’s surface. We also infer that O_2_ is a significant background gas in the atmosphere [6, 34] but its effects on flow and transport are totally unknown. Indeed, the influence of other species (e.g. S_2_ [59], NaCl [38] and Cl [16]) may also be large but are unknown.

McDoniel et al. [39] have modelled how the downwards flow from plumes affect the sublimation atmosphere in specific cases and some understanding of how an anisotropic sublimation-driven atmosphere might interact with volcanic plumes has been achieved through this work. It is, however, a dynamic process. Kosuge et al. [32] have noted that oscillations in macroscopic quantities (density, temperature) may occur because of the interaction.

Volcanic activity is also highly variable [12, 50] and hence by studying the atmosphere in detail we have a chance to see the driving mechanisms and how the system responds to them.

### Mass loss from Io

Io loses ~1 t/s of material to Jupiter’s magnetosphere. The processes involved include atmospheric and surface sputtering, charge-exchange, and photo- and/or electron impact ionization and subsequent pick-up. The relative importance of these processes and how their importance might change with volcanic activity are not well known although we suspect that the Io plasma torus does respond to changes in volcanic activity [7]. It is expected that atmospheric sputtering by co-rotating Jovian magnetospheric plasma dominates and that this produces the Io neutral (“banana”) cloud that accompanies Io in its orbit about Jupiter. Ejection speeds for this process are typically 2–3 km/s. However, evidence of charge-exchange, molecular dissociation and fast neutral products has been found (see review by [62]) and the processes contribute significantly to the total mass loss. They also indicate significant complexity in the interaction.

### The Io-Jupiter interaction

Io’s interaction with Jupiter’s magnetosphere can be divided into two regions [54]. The local interaction (modelled by for example [15]) includes the region around Io itself while the far-field interaction includes Jupiter and its inner magnetosphere. Models of the local interaction have reached a fair degree of sophistication (e.g. [15]) by combining MHD modelling of the plasma interaction with a detailed chemical model but data on the atmospheric densities and pick-up ion rates are needed to provide better constraints. In these models, an ionosphere is formed by electron impact ionization and photoionization and significant electric fields are produced that drive ionospheric electric currents. The currents modify the fields near Io and slow the plasma, directing it around Io. The flow starts to be slowed down some way upstream of the ionosphere and is almost stagnant in the ionosphere itself. The flow past Io’s flanks reaches 1.7 times the speed of the unperturbed flow. Enhanced equatorial UV emissions at the flanks has been observed and models proposed [56]. Downstream of Io, a dense plasma wake is formed with steep temperature gradients. The major questions here revolve around the feedback loops between Io’s atmosphere and the plasma interaction and their response to changes in Io’s volcanic activity. Although we have a conceptual picture of the interaction, the details are vague.

The far-field effects include the production of aurorae at Jupiter. Aurorae are electromagnetic emissions which are connected to the precipitation of magnetospheric plasma onto a planet’s ionosphere. Jupiter’s aurorae have emission features that are associated with its moons [46]. Bright spots appear at the base of magnetic field lines that sweep past Io [10] and are indicative of the strong electromagnetic interaction between Io and Jupiter first recognized by studies of Jovian decametric emissions in the 1950s. The concept of an electric current linking the Io with Jupiter’s ionosphere was put forward by Piddington and Drake [49] and by Goldreich and Lyndon-Bell [20]. The Io “footprint” auroral emissions at Jupiter are at the point where the electrical circuit is closed by currents in and out of Jupiter’s upper atmosphere. The tilted dipole of Jupiter’s magnetic field changes the pathlength of the circuit and also modifies the field strength at Io [9]. The changes propagate by Alfvén waves but there appear to be several effects influencing the brightness of the footprint emissions. These include, in addition to the local interaction at Io, Alfvén wave reflections, magnetic mirroring of the electrons, and kinetic effects close to Jupiter [22]. Recent results from JAXA’s Hisaki spacecraft, launched in 2013, indicate the effects on auroral emissions of varying volcanic activity [61].

### The Io torus

Material removed from Io forms a neutral cloud which surrounds it and accompanies it in its orbit about Jupiter. This cloud undergoes electron impact ionization and charge-exchange to produce a dense plasma (up to 4000 electron cm^−3^) called the Io plasma torus. Studies of the neutral cloud and the Io plasma torus reveal it to contain not merely sulphur, oxygen, and SO_2_ but also sodium, potassium, and even chlorine [35]. At present limits on other species are not tight enough to rule out non-negligible amounts of other alkali metals, halogens, nitrogen, and silicon and the detection of dust [33] suggests that less volatile species may also be present. The torus itself has been reasonably well characterized through visible and UV emissions although the distributions of neutrals such as atomic sulphur and SO_2_ that feed the torus remain somewhat poorly known observationally. Models tend to assume that sodium is an adequate proxy despite it only contributing around 5% to the total torus density. The torus participates in the interaction with Io itself and there remain numerous questions about how the feedback between plasma generation at Io and the Io torus density is stabilized. This illustrates also the importance of combining a detailed investigation at Io with a more synoptic view of the system which can be given by UV spectrometers in Earth orbit and by ground-based monitoring of the large scale plasma and neutral distributions. Studies of the Io plasma torus have been conducted by Hisaki which carries an EUV spectrometer for spectral imaging of the EUV emissions (e.g. [66]). Most of the energy loss from the torus (around 10^12^ W) comes through these EUV emissions. Typical spectra of Io show neutral emissions from near the moon as well as contributions from plasma torus ions.

The idea that variations in volcanic activity lead to changes in the torus emissions might seem obvious but a clear, unambiguous demonstration still needs to be made. The whole concept with respect to Io’s interaction provokes additional questions such as “What types of eruptions are needed to enhance the neutral and plasma clouds?” or “Are there geographical requirements on the position of volcanic activity that are needed to influence the mass loss?”

The major issues connected to the torus itself revolve around its energy budget. Pick-up of ions by Jupiter’s corotating field extracts energy from Jupiter’s rotation which is then lost via emissions and charge-exchange processes. However, it has long been known that the torus is warmer than simple chemical balances would suggest unless a hot electron component is added to the mix. The energy picked up by ions can only fuel 34–74% of the observed radiation [14]. An influx of electrons heated in the outer magnetosphere has been proposed [63] and this continues to be assumed as the origin of the required additional energy source (e,g, [66]).

### The influence of Iogenic material on Jupiter and other moons

Once ions are picked up by the Jovian magnetic field they slowly diffuse throughout Jupiter’s magnetosphere and dominate its plasma population [52]. The diffusion rate is known roughly but the mechanisms involved are still unclear. On reaching the outer magnetosphere, processes which again are only poorly understood lead some of the ions and electrons to precipitate along field lines to form Jupiter’s prominent aurorae that are distinct from the aurorae more closely associated with the Galilean moons. Charge-exchange processes are also prevalent and produce fast neutrals which, unless a further collision occurs (with a satellite for example), leave the system.

The diffusing ions and fast neutrals can impact the surfaces of all of the solid bodies in the Jovian system. The ions, neutrals, and, perhaps importantly, the electrons produce a charged particle bombardment of Europa’s surface which can itself lead to interesting processes as shown in laboratory experiments (e.g. [18] cf. [58]). For example, ion and neutral implantation in Europa’s surface ices provides additional species which can react with endogenic material. Hydrated alkali sulphates and chlorides are potential products. CO_2_ has been observed as a gas trapped in ice on both Ganymede and Callisto. CO_2_ has been seen as an atmospheric constituent on Callisto [8]. If these species can be released by energetic particle impact and transported to Europa, we have a potential source of carbonates and complex hydrocarbons.

As pointed out by Johnson et al. [26], these products will not remain at the surface for long. Upwelling of liquid water will “wash” this material off the surface and transport it into any sub-surface ocean. Subduction processes and re-surfacing will also occur. Meteoroid bombardment will increase this rate. If the ocean is within a few kilometres of the surface, it is inconceivable that these potential reactants (nutrients?) would not reach the ocean on fairly rapid timescales. Hence, even if Europa had started as a rocky core with a pure water ice mantle, it is now “contaminated” with elements which can combine to produce long chain molecules and perhaps more.

Irradiation of water ice can also result in the production of hydrogen peroxide and molecular oxygen through trapping of the hydroxyl radical [26]. O_2_ has in fact been detected in Europa’s atmosphere indirectly using HST through observation of the OI emission at 1304 Å [21]. The importance of Io in this game of celestial pin-ball should not be underestimated and understanding the source and distribution of Iogenic material within the Jovian system may have implications for objects that are perceived as having potential for past and present life.

## Objectives of a detailed study of Io

NASA’s Decadal Survey in 2011 recognized the importance of an Io Observer mission and outlined 8 objectives as shown in Table 1. Those indicated in green were identified as primary objectives with those in yellow defined as secondary. These objectives are sufficiently comprehensive and broad that they still cover many of the goals needed to be addressed by a future programme to Io. However, it is worthwhile going a little deeper.

**Table 1:**
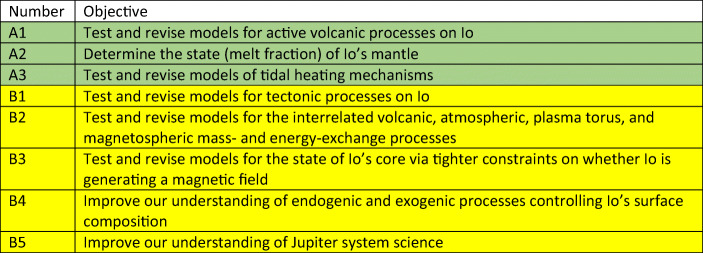
Objectives for an Io Observer mission taken from the Decadal Survey in 2011. (Vision and Voyages for Planetary Science in the Decade 2013–2022, 2011; ISBN 978–0–309-22,464-2.)

Within the context of proposals for the Io Volcano Observer, the proposers suggested “**Follow The Heat**” as a theme for the mission [41]. Essentially they focussed on the looking at energy flow from tidal dissipation to energy loss processes through volcanic and thermal emissions. However, Io can also be looked at by following the mass – how material is transported from the interior to the magnetosphere and beyond. These two themes are complementary and are intellectually “satisfying” as they are essentially based around fundamental conservation laws. In Europe, “**Follow The Mass**” may have a greater resonance with the community because of the relative strength of European space plasma physics. However, addressing both elements are of equal importance. McEwen et al. [40], in an abstract which included several European scientists as co-authors, gave a set of 23 objectives for such a mission and we provide an update and re-categorization of those objectives in Table 2. We have also included objectives based upon a series of open questions about the atmosphere-magnetosphere interaction provided by Bagenal and Dols [2]. By combining these ideas, we can derive a set of objectives that are both scientifically strong and achievable through fairly traditional methods.

**Table 2:**
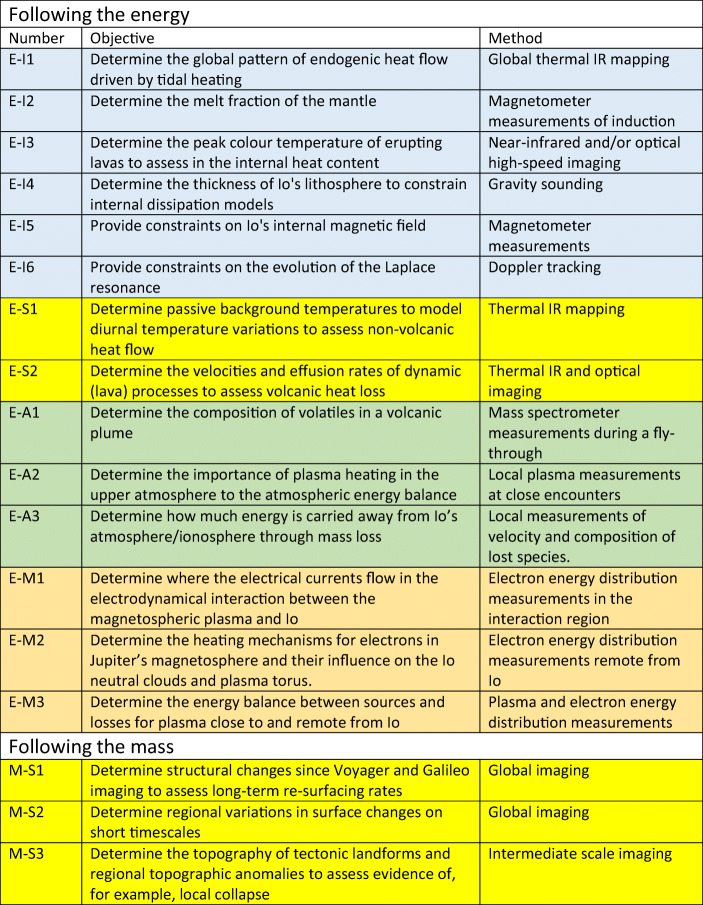

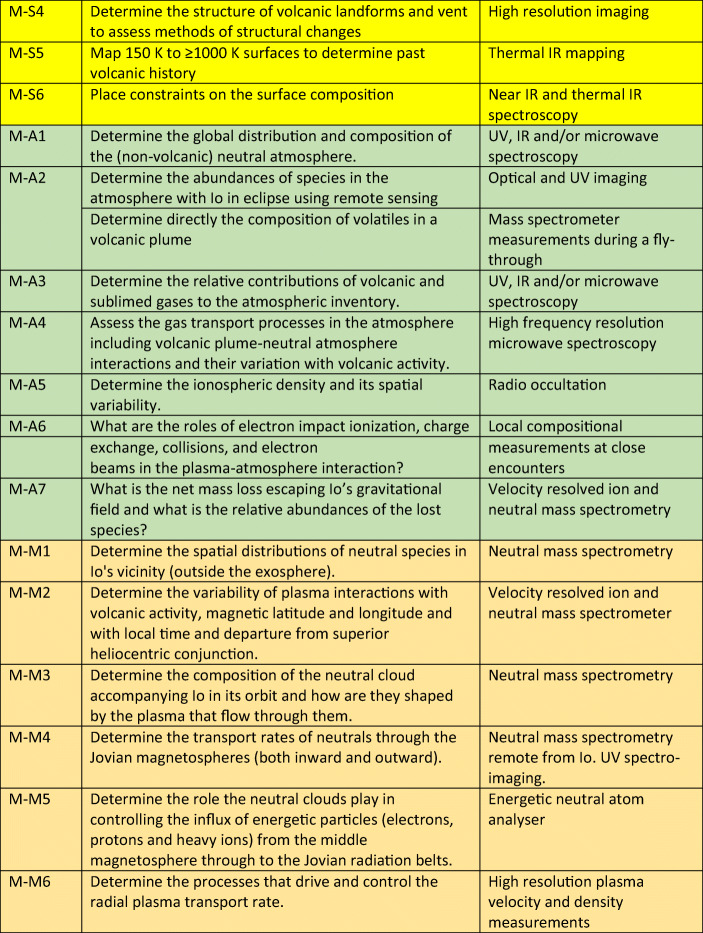
Objectives for a mission to Io categorized according to two themes - summing the energy and summing the mass. The objectives are identified according to I (interior -blue), S (surface - yellow), A (atmosphere - green), and M (magnetosphere - orange). Many objectives are modified versions of those originally presented in McEwen et al. [40] and by Bagenal and Dols [2].

## Previous and forthcoming missions

The Galileo mission was planned as the first giant planet orbiter and the first mission to send a probe into the atmosphere of a giant planet. It should have capitalised on the success of the Voyagers. As is well known, however, the mission did not run smoothly. It was delayed as a result of the loss of the Space Shuttle in Challenger in 1986 and when launched in 1989 the high gain antenna failed to open and the data rate from Jupiter was reduced to only 100 bit/s. These events had several consequences. Inevitably some of the experiments onboard were outdated. The plasma sciences experiment, for example, did not provide significant improvements over our knowledge from Voyager. The low data rate certainly affected the return from the major data rate consumers (the camera and the infrared spectrometer) reducing coverage enormously despite efforts to improve the onboard data compression and extensive use of the DSN’s 70 m network.

The Cassini fly-by in December 2000 has provided much complementary information about the Jovian system. The remarkable UV spectrometer observations of changes in the Io plasma torus over a 100 day period are an excellent example. But the fly-by was a relatively distant one, so that the most interesting observations of the system were remote sensing time series at lower spatial resolution and in situ magnetospheric measurements in the distant magnetosphere. Similarly, New Horizons made a distant fly-by but did make major contributions to, for example, studies of Io in eclipse.

This is not to belittle the previous missions to the Jovian system - they have clearly brought major steps forward in our knowledge – but there remain many open questions which have been summarized in Bagenal et al. [3].

In the US, teams have come together on several occasions to propose missions to Io within the Discovery programme which go a long way towards responding to the Decadal Survey recommendations. These proposals have performed extremely well in the selection process. In the 2014 round, the Io Volcano Observer (IVO) proposal was ranked category 2 (selectable but viewed as being of lower priority than category 1) indicating that NASA’s review concluded that the mission was feasible within the Discovery programme constraints. The mission has been re-proposed for the current (2019) Discovery round with a multi-national team including contributions from Germany and Switzerland and a slightly modified focus. It has been selected as one of the last 4 missions in the competition of which one or two will be selected in 2021 following an 18 month study phase. Clearly, selection of IVO for Discovery would require re-evaluation of the content of this White Paper. A European mission to Io focusing on the flow of mass through the Jupiter system using detailed plasma instrumentation (rather than looking directly at Io itself as IVO currently does) could be an option. On the other hand, if IVO were not to be selected, alternative configurations with ESA as a partner might be considered with the New Frontiers programme being a possible option.

## Relationship to other ESA missions

A mission to Io would complement ESA’s existing programme well. For example, Fritz Neubauer (Giotto magnetometer PI and leading magnetospheric physicist) was often fond of saying that Io’s interaction with Jupiter’s magnetosphere was almost comet-like and a study of the interaction of Io with the plasma torus will undoubtedly attract large parts of the Rosetta plasma community. An Io mission also requires instrumentation that can study high surface temperatures and is thus related to BepiColombo Mercury Planetary Orbiter objectives. The need for thermal mapping of high temperature flows on Io would be a natural extension of the studies to be conducted at Mercury. The study of volcanic flows on Io could also provide input to the interpretation of ancient volcanic flows on Mercury. Should ESA become engaged in investigations of the Moon, studies of flood lavas on Io could provide present currently active analogies to processes that occurred on the Moon more than 3 billion years ago.

The most obvious connection is to the JUICE mission. Iogenic material peppers the surfaces of Ganymede and Europa. Hence we observe with JUICE the influence of this material on the surfaces of the outer Galilean satellites but the actual source of this material is not well documented. Consequently a detailed investigation of the source would be highly complementary.

## International interest

The interest in investigating, in detail, the physical processes associated with Io is immediately evident when one discusses such an idea both with colleagues in the Solar System community and the general public. The excitement a mission to Io generates is also evident in the fact that an “Io Observer” is regularly included in NASA’s long-term planning. For example, the last decadal survey included Io as a potential candidate for the New Frontiers programme (as NF-5). Proposals to NASA’s Discovery programme have now been made on 3 occasions. Several of the ideas included within these missions and mission proposals can inform an ESA-based approach.

The Extreme Ultraviolet Spectroscope for Exospheric Dynamics on the Japanese Hisaki mission is dedicated to observations of Io’s EUV emissions (e.g. [23, 30, 31]) and it could be easily imagined that JAXA would like to follow-up on the success of their mission with participation in an in situ mission.

## Mission profiles

It is no surprise that the major engineering challenge in flying a mission to perform a detailed study of Io comes from defending the spacecraft from radiation. It is also apparent from studies that orbiting Io for any length of time would require protection against Mrad levels of total ionizing dose (TID). Although the densities of 50 keV- 50 MeV ions near Io’s orbit are two orders of magnitude lower than at Europa and electron fluxes between 1 and 100 MeV are comparable, Io probably has a harsher high energy electron environment than Europa. Hence, defending the spacecraft might be possible but would require study. On the other hand, proposal work within the NASA system [1] has shown that the TID can be kept below 200 krad if flybys of Io are performed from an orbit about Jupiter that is inclined by around 45 degrees to the Jovian equatorial plane. The fly-bys would be typically at ~18 km s^−1^ and roughly orthogonal to the Io orbit plane. We would suggest, following the IVO planning, that 10–20 flybys of the moon, at various distances, lo longitudes, and orbital positions of Io with respect to Jupiter would be sufficient to address many of the objectives discussed here.

We therefore see two means of implementing an Io mission, each with sub-categories. This is summarized in Table 3. An Io orbiter mission is most desirable but also the most challenging because of the radiation environment. Clearly, ESA could attempt to implement an Io mission as an ESA-only L-class mission. We believe that such a mission would serve a larger community than the current BepiColombo mission to Mercury and would receive significant support from the planetary sciences, the magnetospheric physics, and possibly the exoplanet astronomy communities. At L-class, a Japanese collaboration could be considered and JAXA has shown interest in the Io-Jupiter interaction in past missions. However, given that this region of the Solar System is already the subject of one L-class mission, it would not seem prudent to propose another at this time. Hence, we consider other options.

**Table 3:**
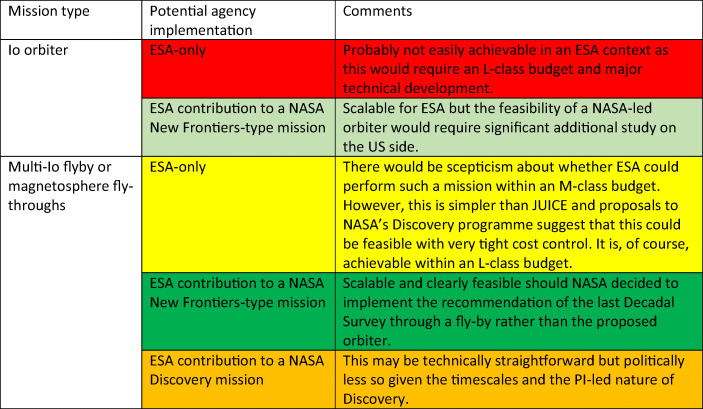
Methods of implementing a mission to the Io system with an ESA framework. The missions are colour-code (from red being most challenging through orange and yellow to green being the most straightforward).

For NASA, an Io orbiter is clearly beyond a Discovery-level budget. Hence, an ESA contribution to a New Frontiers proposal would appear to be the preferred alternative for an Io orbiter but studies would be needed to assess what ESA could contribute given the harsh radiation environment.

The Discovery proposals and their evaluations as being selectable illustrates that multi-Io flyby missions can be achieved with budgets of <500 M$ in a US context. Given the overhead associated with ESA’s missions (development duration, satisfying geo-return etc.) this is probably at the margin of what is feasible for ESA within an M-class budget. However, ESA will have gained considerable experience of working in the Jupiter system through the JUICE programme and we re-iterate that radiation issues for an Io multi-flyby mission can be less than those to be encountered by JUICE. Hence, we believe that an ESA-only approach should not be automatically rejected. ESA’s discussions with NASA have indicated the difficulties of making a contribution to a NASA Discovery class mission and hence this is probably off-the-table at least in the short term. A contribution to a NASA New Frontiers mission of this type would, on the other hand, be straightforward. Consequently, if IVO is ultimately rejected in the forthcoming Discovery selection procedure, a New Frontiers contribution could prove to be a viable option.

We also note in passing that the re-establishment of the International Jupiter Watch (which was modelled on the International Halley Watch and has been essentially defunct for the past 15 years or so) as a complement to any Io mission would be beneficial to several of the objectives.

## Io within a more general theme

The above has described a rather specific mission and goals for that mission. However, Io could be seen as an important element within larger themes.

Io is one of several major objects in our Solar System that are characterized by dynamic phenomena. These include Enceladus, Triton, Europa, and possibly Venus and Titan. Describing a theme connected to current dynamic and evolutionary phenomena would place these objects as natural targets for future missions (i.e. The investigation of current dynamic processes in our Solar System). Alternatively, the processes we now see on Io are probably related to past evolutionary processes on objects such as the Moon. Hence, Io could also fall under a theme linking past and present phenomena (i.e. Evolutionary processes on ancient objects by studying present-day analogues). In both cases, Io could become a key element within a larger objective.

## Conclusion

The Galilean moon, Io, is one of the most fascinating objects in our Solar System with a vast array of, relatively poorly understood, phenomena of interest to a wide ranging community. When investigating Io, we are looking at a vast array of geophysical, geological, geochemical, and dynamical interactions. The active processes on the surface of Io today are similar to those that formed the surfaces of the terrestrial planets up to billions of years ago and the influence of tidal heating (with the associated coupling to the orbit) may be similar to that in exoplanetary systems that leads to extension of their habitable zones. Hence, Io is an important inter-disciplinary target of research.

We have presented a scientific justification for such a mission and assert that a dedicated mission to the Io-Jupiter system is long overdue. The latest NASA Discovery proposal for Io sought to implement a mission by using “Follow The Heat” as a theme. On the other hand, mass transport is also fundamental and using this as a theme may be more in keeping with the current structure of the European community with its strength in magnetospheric physics. Hence, we see a more comprehensive goal of “Investigating Io by Tracing Mass and Energy” as something we can aspire to.

Following JUICE, ESA should have the technical capabilities to provide spacecraft elements for a contribution to a NASA New Frontier-type mission which could provide a highly complementary programme for NASA and ESA scientists. Despite scepticism, it is not inconceivable that ESA could produce a stand-alone multi-flyby mission to Io and we believe this should be studied also as a means of identifying mission components that ESA could offer for a future New Frontiers call.

The remarkable volcanic nature of Io will be a real magnetic for the general public and we would expect a mission to provoke huge interest in the determination of the physical processes occurring on and around this remarkable object. A detailed investigation of Io is now overdue.

## Data Availability

No data used.
